# M1 Macrophages Are More Susceptible to Necroptosis

**DOI:** 10.33696/immunology.3.084

**Published:** 2021

**Authors:** Qin Hao, Steven Idell, Hua Tang

**Affiliations:** Department of Cellular and Molecular Biology, The University of Texas Health Science Center at Tyler, Tyler, Texas, USA

**Keywords:** Macrophage polarization, M1/M2 phenotypes, Necroptosis, Inflammation, RIPK3, MLKL, ZBP1

## Abstract

Macrophages play a crucial role in host innate immune defense against infection and tissue injury. Although macrophage activation and polarization has been well studied, we know less regarding the role of macrophage activation/polarization in inflammation-associated necrotic cell death. By using bone marrow-derived macrophages, we have recently demonstrated that M1 macrophages are much more susceptible than M0 and M2 subtypes of macrophages to necrotic cell death. Moreover, we showed that the enhanced necroptosis in M1 macrophages is dependent on the kinase activity of receptor-interacting protein kinase-3 (RIPK3) and may involve the upregulation of key necroptosis signaling molecules including RIPK3, mixed lineage kinase domain-like protein, and Z-DNA/ RNA binding protein 1. Our findings provide novel insights into the mechanisms of M1 macrophage engagement in inflammation and tissue injury.

## Macrophage Phenotypes

Macrophages are important cells of the innate immune system and play a crucial role in host immune defense against infection and injury [1–3]. Macrophages form the first line of defense against airborne particles and microbes through multiple functions including phagocytosis, production of cytokines and chemokines, and antigen presentation. Macrophages are highly plastic cells and their phenotypes and functions can be regulated by the local microenvironment. Depending on the context, macrophages can be activated and polarized into different subsets. Macrophage polarization is a process whereby macrophages mount a specific phenotype and a functional response to the surrounding stimuli. Two major macrophage sub-populations with distinct functions have been characterized, and they are the M1 (also termed classically activated or inflammatory) and M2 (alternatively activated or anti-inflammatory) macrophages [2–4]. M1 macrophages are typically induced by pathogen-associated molecular patterns, such as double-stranded RNA (dsRNA) and lipopolysaccharide (LPS), and by Th1 cytokines including interferon γ (IFNγ) and tumor necrosis factor α (TNFα). The activated macrophages acquire transcriptional changes to produce higher levels of pro-inflammatory cytokines and chemokines as well as reactive oxygen species, through which they contribute to host defense against pathogens and tissue damage [3,4]. On the contrary, M2 macrophages can be induced by Th2 cytokines such as interlleukin-4 (IL-4) and IL-13 as well as anti-inflammatory cytokines IL-10 and transforming growth factor β (TGFβ). M2 macrophages are anti-inflammatory and implicated in tissue repair, remodeling and vasculogenesis. M2 macrophages can be further divided into four different subsets that consist of M2a, M2b, M2c and M2d, depending on the stimuli received [5,6].

## Necroptosis: A Critical Regulator of Inflammation

Programmed and regulated lytic cell death such as necroptosis is increasingly recognized as a driving factor in the pathogenesis of various forms of tissue injury and inflammation resulting from viral and bacterial infections, sepsis, trauma, sterile inflammation, mechanical ventilation, ischemia-reperfusion, and blood transfusion [7–13]. Unlike apoptosis, necroptosis causes cell membrane rupture, which triggers and amplifies inflammation thorough the release of damage-associated molecular patterns, such as high-mobility group protein B1, IL-1 family cytokines, nucleic acids, as well as S100 proteins [11,14]. Necroptosis is initiated by receptor-interacting protein kinase-3 (RIPK3) and executed by the effector mixed lineage kinase domain-like protein (MLKL) [9,15]. Activation of RIPK3 induces MLKL phosphorylation and membrane translocation and subsequent disruption of the plasma membrane, leading to necrotic cell death [16–19]. Necroptosis is negatively regulated by caspase-8 together with a caspase-like molecule c-FLIP_L_ [20] and by the ubiquitin ligases cIAP1 and cIAP2 [21]. Thus necroptosis is sensitized under caspase inhibition and cIAPs for degradation by Smac mimetics [19]. Caspase 8 inhibitors have been identified in murine cytomegalovirus and herpes family viruses [22], while loss of cIAPs can happen during cytokine stimulation [23]. Necroptosis can be induced by TNF superfamily death ligands, Toll-like receptor-3 (TLR3) and TLR4 ligands when caspases are inhibited and/or cIAPs are degraded [15–19,24]. TNF-induced necroptosis requires the complex formation of RIPK1-RIPK3-MLKL [19,25]. Direct phosphorylation of RIPK3 by RIPK1 has not been demonstrated; hence oligomerization of RIPK3 driven by the RHIM domains of RIPK1 and RIPK3 leads to RIPK3 autoactivation [26]. Consistent with that, RIPK3 can be activated by associating with other two RHIM-containing adaptor proteins such as TRIF and Z-DNA/RNA binding protein 1 (ZBP1; also known as DAI/DLM-1) to mediate necroptosis by TLR3/4 activation and virus infection, respectively [24,27,28]. RIPK3 activation is thus regulated by the competitive interactions with other three RHIM-containing proteins including RIPK1, TRIF and ZBP1 [9,15,24,29]. It has been shown that serum levels of RIPK3 are elevated in critically ill patients and are associated with the development of acute respiratory distress syndrome in sepsis, trauma and COVID-19 infection [30–32]. Recent experimental studies indicate that inhibition of necroptosis confers protection against acute lung injury and inflammation induced by LPS [33–35], hyperoxia [36], mechanical ventilation [37], sepsis/systemic inflammatory response syndrome [38–40], respiratory syncytial virus Infection [41], bacterial pneumonia [42–44], trauma [45], and blood transfusion [46]. Hence, the targeting of necroptosis holds significant promise for the treatment of acute lung injury and inflammation.

## M_1_ Macrophages Are More Susceptible to Necroptosis

Although macrophage activation and polarization has been well studied [2–4], we know less regarding the role of macrophage activation/polarization in inflammation-associated necrotic cell death. The macrophage subtypes that are susceptible to necroptosis are not clear and the underlying mechanisms are likewise poorly understood. Most necroptosis studies are performed in resting cells [15–19,24], which is a commonly used approach to define the necroptosis signaling pathway. As inflammation and tissue injury is often associated with release of cytokines among other mediators, innate immune cells such as macrophages and tissue structural cells are expected to encounter these pro-inflammatory effectors and be activated. Based on the idea, in our recent study, we pretreated bone marrow-derived macrophages with M1 (LPS, dsRNA and IFNγ) or M2 (IL-4, IL-10 and TGFβ) macrophage subtype inducers and then investigated the subtype-dependent responses to different necroptosis inducers. We found that macrophage necrotic cell death and the releases of lactate dehydrogenase and dead cell proteases were greatly augmented in M1 but not M2 macrophages, and the enhanced effects were blocked by two structurally distinct specific RIPK3 inhibitors GSK872 or GSK843 [47]. Our findings clearly demonstrate that M1 but not M2 subtypes of macrophages are much more susceptible to inflammation-related necrotic cell death in a RIPK3 kinase activity-dependent manner. The lytic cell death of M1 macrophages can result in release of not only damage-associated molecular patterns that are normally seen in resting cells [11,14], but also of high levels of newly synthesized pro-inflammatory cytokines and chemokines [3]. We thus posit that the burst of such immune-stimulatory intracellular components could trigger and amplify inflammation, form a pro-inflammatory cycle and ultimately contribute to the pathogenesis of acute tissue injury and inflammation. Recent evidence has shown that necroptosis of alveolar macrophage plays an important role in the pathogenesis of acute lung injury and inflammation [41,48]. Our edifying findings also suggest that, like M1 macrophages, LPS-, dsRNA- or IFNγ-activated/primed tissue structural epithelial and endothelial cells could be susceptible to necroptosis, which merits further investigation. In contrast, the M1 macrophage inducers did not enhance macrophage susceptibility to apoptosis inducers [47].

**Figure 1:**
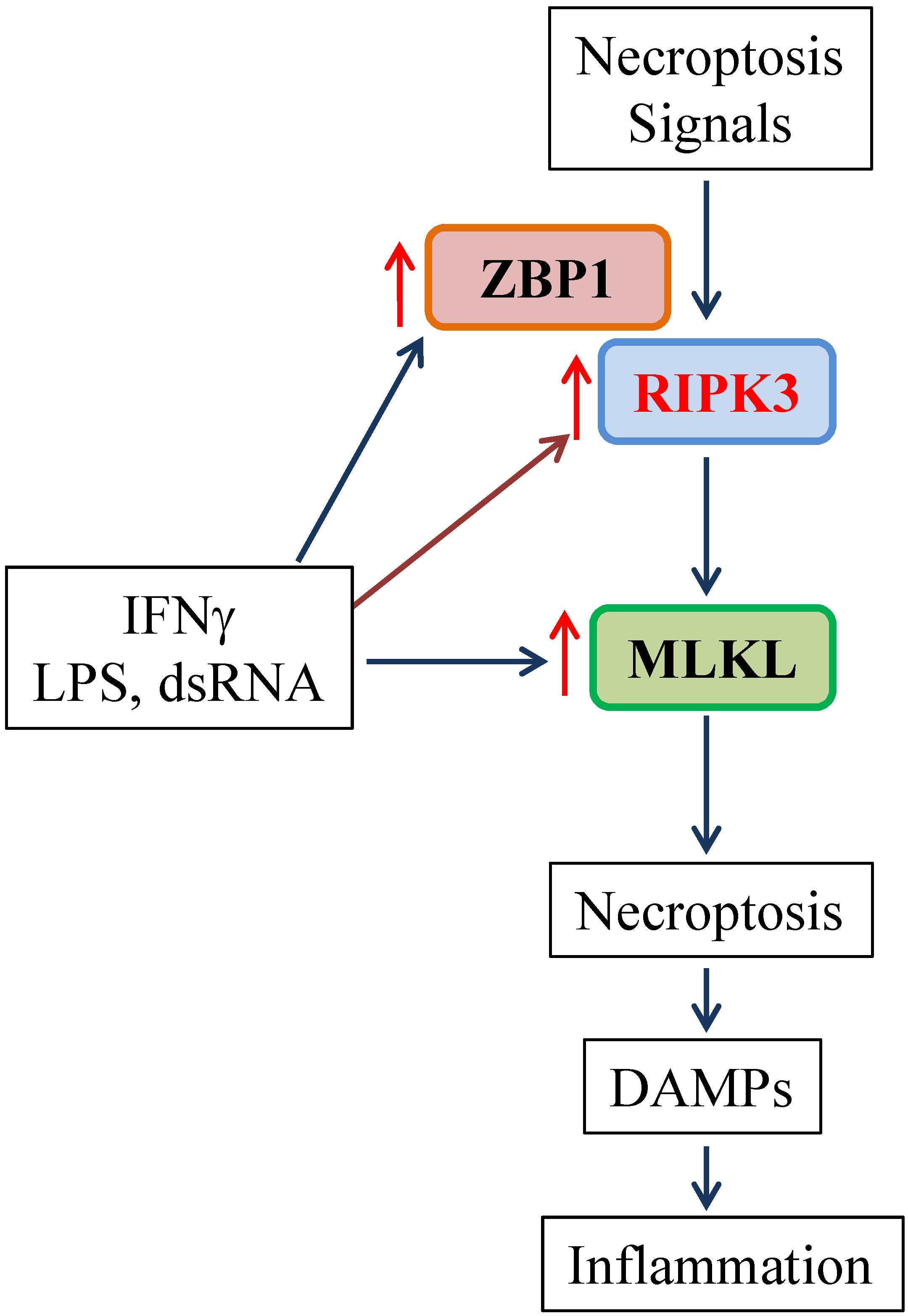
Potential mechanism of the enhanced necroptosis in M1 macrophages. DAMPs: Damage-associated Molecular Patterns.

Delineation of the mechanisms participating in M1 macrophage necroptosis may offer a novel strategy to control aberrant host innate immune responses and tissue damage. Mechanistically, we found that the necroptosis effector MLKL and the key necroptosis signaling molecule ZBP1 were exclusively induced by M1 but not M2 macrophage subtype inducers [47] (Figure 1). We also found that the protein but not mRNA levels of RIPK3 were upregulated in M1 macrophages, which suggests that protein synthesis or posttranslational regulation (e.g. stability) may be involved in the upregulation of RIPK3 protein. Thus, enhanced necrotic cell death occurring in M1 macrophages may likely attribute to the upregulation of key necroptosis signaling molecules including RIPK3, MLKL and ZBP1 (Figure 1). In addition, we found that these three necroptosis signal molecules were readily upregulated in M1 macrophage inducer-primed dendritic cells [47]. These results suggest that like M1 macrophages, the activated dendritic cells could be susceptible to necroptosis, promoting antigen presentation to T lymphocytes as demonstrated previously [49]. More studies are needed to define the proposed mechanisms.

## Conclusions and Perspectives

By using bone marrow-derived macrophages, we have recently reported that that M1 macrophages induced by LPS, IFNγ and dsRNA were much more sensitive than M0 and M2 subtypes of macrophages to various necrotic cell death inducers. The enhanced necroptosis in M1 macrophages is dependent on RIPK3 kinase activity and may involve the upregulation of key necroptosis signaling molecules including RIPK3, MLKL and ZBP1 (Figure 1). These findings provide novel insights into the mechanisms of M1 macrophage engagement in inflammation and tissue injury. Since we used bone marrow-derived macrophages for the study, it will be important to determine if M1 macrophages are more susceptible to necroptosis *in vivo* under disease conditions. Although inhibition of necroptosis confers protection against tissue injury and inflammation, the role of macrophages in the process is not clear. Generation of macrophage-specific knock out of RIPK3 will enable us to address this gap in current knowledge and to identify a specific therapeutic target that could be further developed to control various forms of inflammation and tissue injuries, in which macrophage death is a common feature.
